# Parenteral Treprostinil as an Effective Bridge to Lung Transplant in Patients with Severe Pulmonary Hypertension Associated with Interstitial Lung Disease

**DOI:** 10.3390/jcdd13090460

**Published:** 2026-09-12

**Authors:** Jennifer Hopkins, Shameek Gayen

**Affiliations:** 1 Department of Internal Medicine, Temple University Hospital, Philadelphia, PA 19140, USA; 2 Department of Thoracic Medicine and Surgery, Lewis Katz School of Medicine, Temple University, Philadelphia, PA 19140, USA; shameek.gayen@tuhs.temple.edu

**Keywords:** pulmonary hypertension, interstitial lung disease, treprostinil, prostacyclin analog, lung transplant, veno-arterial extracorporeal membrane oxygenation

## Abstract

Pulmonary hypertension associated with interstitial lung disease (PH-ILD) is associated with substantial morbidity and mortality. Lung transplantation remains the definitive therapy for selected patients; however, severe pulmonary vascular dysfunction and hemodynamic instability may complicate transplant candidacy and pre-transplant management. The role of parenteral prostacyclin therapy as a bridge to transplantation in this population remains poorly defined. The primary objective of this retrospective case series was to evaluate the utility of parenteral treprostinil as a bridge therapy for lung transplantation in 14 patients with severe PH-ILD from 2018 to 2025. Secondary objectives were to describe subsequent changes in clinical outcomes, including hemodynamic parameters measured by right heart catheterization, echocardiographic findings, the six-minute walk distance, oxygen requirements, the need for veno-arterial extracorporeal membrane oxygenation (VA-ECMO), and successful lung transplantation. Parenteral treprostinil was associated with significant improvements in pulmonary artery systolic pressure, mean pulmonary artery pressure, pulmonary vascular resistance, and BNP levels, while pulmonary capillary wedge pressure, cardiac output, cardiac index, and oxygen requirements remained stable following therapy initiation. Echocardiography demonstrated improvement in right ventricular dilation and right ventricular dysfunction. Most patients were successfully bridged to lung transplantation, while only two patients died prior to transplantation. In selected patients with severe PH-ILD, parenteral treprostinil may improve pulmonary hemodynamics and facilitate a successful bridge to lung transplantation without worsening oxygenation.

## 1. Introduction

Pulmonary hypertension associated with interstitial lung disease (PH-ILD) represents a severe condition in patients with significant morbidity and mortality. Lung transplantation remains the definitive treatment for selected patients with end-stage disease; however, pre-transplant management is often complicated by severe pulmonary vascular disease, hemodynamic instability and the need for extracorporeal support.

The management of severe PH-ILD remains challenging due to limited therapeutic options and poor outcomes with pulmonary hypertension therapies in this population. Parenteral prostacyclin therapies, such as intravenous or subcutaneous epoprostenol and Treprostinil, are currently used for the treatment of Group 1 pulmonary arterial hypertension (PAH) by promoting the vasodilation of the pulmonary and systemic vasculature and inhibiting platelet aggregation [1]. Parenteral prostacyclin therapies have shown hemodynamic and mortality benefits in patients with decompensated right ventricular failure or New York Heart Association (NYHA) functional class IV symptoms [2]. For patients with PH-ILD, inhaled treprostinil has demonstrated a benefit with improvement in exercise capacity, shown by improved six-minute walk tests over a 16-week period and remains the only Food and Drug Administration (FDA) approved therapy for Group 3 PH, specifically PH-ILD [3].

Data regarding parenteral treprostinil in patients with severe PH-ILD disease remain limited while parenteral epoprostenol has shown improvements in right ventricular hemodynamics in patients with Group 1 PAH [2]. Compared to intravenous epoprostenol, both subcutaneous and intravenous treprostinil have a longer half-life (four hours vs. 3–5 min), greater chemical stability at room temperature, and can also be administered via pumps, which prevents the need for central lines and may reduce associated central line infections [4]. Concern regarding ventilator-perfusion mismatch and hypoxemia may limit the use of parenteral treprostinil in PH-ILD disease. However, in Group 1 PAH, parenteral treprostinil has been shown to improve 6 min walk tests and improve cardiopulmonary dynamics [5]. Therefore, the objective of this case series was to evaluate the safety and efficacy of parenteral treprostinil therapy as a bridge to lung transplantation in patients with severe PH-ILD.

## 2. Materials and Methods

This was a retrospective case series examining adults with severe pulmonary hypertension associated with interstitial lung disease (PH-ILD) who were treated with parenteral treprostinil therapy. The study was conducted at a single tertiary care lung transplant center between July 2018 and March 2025. Data were collected retrospectively from electronic medical records.

### 2.1. Study Population

Patients were screened based on the following inclusion criteria: age older than 18 years, a confirmed diagnosis of interstitial lung disease, a confirmed diagnosis of pulmonary hypertension from right heart catheterization (RHC) hemodynamics defined as a mean pulmonary artery pressure greater than 20 mmHg, pulmonary capillary wedge pressure (PCWP) less than or equal to 15 mmHg, and pulmonary vascular resistance (PVR) greater than or equal to 3 Woods units (WU), and treatment with parenteral treprostinil therapy. Exclusion criteria were patients diagnosed with pulmonary hypertension World Health Organization (WHO) Groups 1, 2, 4 and pulmonary hypertension associated with chronic obstructive pulmonary disease as determined by an obstructive pattern —Forced Expiratory Volume/Forced Vital Capacity (FEV1/FVC) ratio less than 70%- on pulmonary function testing (PFTs).

The choice of parenteral therapy over inhaled alternatives was determined by individual pulmonologists based on medication tolerability and the severity of hemodynamic disease at the time of initial right heart catheterization (RHC). The patients included in this study had severe group 1 PAH physiology and were deemed too decompensated for inhaled prostacyclin therapy; therefore, parenteral therapy was chosen at the discretion of the treating pulmonologist.

The initial dose of parenteral treprostinil was started at 1.25 ng/kg/min either by continuous subcutaneous or intravenous infusion. Titration was determined by increasing the rate of infusion, ranging from 0.625 to 2.5 ng/kg/min per week for the first four weeks, then by 2.5 ng/kg/min per week for the subsequent duration if the medication was well tolerated. Subjects were initiated on treprostinil in the inpatient setting; dose titration was done under careful monitoring, and the standard dose increase was 1.25 to 2.5 ng every 12 to 24 h. Subjects underwent inpatient initiation first with rapid up titration of IV treprostinil then, once on a stable dose, were discharged on subcutaneous treprostinil. One patient initiated subcutaneous treprostinil in the outpatient setting without initial intravenous titration. If a patient experienced adverse effects while on parenteral Treprostinil, the rate of the titration was slowed. The maximum dosage of therapy ranged from 20 to 80 ng/kg/min and was determined by the treating pulmonologist.

Variables collected included demographic information, ILD subtype, additional pulmonary vasodilator therapies, ILD treatment including systemic corticosteroids and steroid-sparing immunosuppressive agents, right heart catheterization parameters, echocardiography hemodynamics such as right ventricular dilation and dysfunction, pulmonary functions tests, 6 min walk test (6MWT), distance and oxygen requirements, the use of extracorporeal membrane oxygenation (ECMO), and survival outcomes.

The use of VA-ECMO was determined by a multidisciplinary team consisting of specialists in cardiothoracic surgery, pulmonology, critical care, pulmonary hypertension, and cardiology. ECMO was used in patients with decompensated right ventricular failure, progressive hypoxemia, or hemodynamic instability despite maximal medical therapy.

Lung transplant outcomes including successful transplantation, time to transplantation, and the presence/duration of ECMO support as a bridge to transplant success were assessed.

Our study met with approval for a waiver of informed consent according to the Western Institutional Review Board (protocol #31795). Procedures were followed in accordance with the ethical standards of the Western Institutional Review Board and the tenets of the Helsinki Declaration of 1975.

### 2.2. Primary Outcome

Our primary outcome was the change in hemodynamic parameters measured via right heart catheterization and transthoracic echocardiography (TTE) from baseline to after parenteral treprostinil initiation. On TTE reports, echocardiographic improvement was defined as a reduction in the severity of right ventricular dilation and dysfunction, graded categorically as normal, mild, moderate or severe. RV dilation was a subjective measurement, with normal right ventricular size defined as less than or equal to two-thirds the size of the left ventricle on the four-chamber apical view on echocardiography [6]. RV dysfunction was determined by a combination of tricuspid annular plane systolic excursion (TAPSE) and visual estimation by a cardiologist at the institution.

### 2.3. Secondary Outcome

Secondary outcomes included changes in functional capacity measured by the six-minute walk test (6MWT) distance and oxygen requirements. Clinical and transplant outcomes, including successful lung transplantation rates, time to transplantation, the requirement for and duration of VA-ECMO support, and overall survival outcomes.

### 2.4. Statistical Analysis

Statistical analysis was done using Microsoft Excel Version 16.101.3 and SPSS Version 29. Data normality was assessed using the Shapiro–Wilk test. Because the hemodynamic variables demonstrated a non-normal distribution, data were expressed as medians accompanied by interquartile ranges (IQR; 25th–75th percentiles) rather than means and standard deviations. To evaluate the effect of parenteral treprostinil on right heart catheterization hemodynamic measurements, a Wilcoxon signed-rank test was performed. Two-tailed *p*-values less than 0.05 were considered statistically significant. We conducted statistical analysis of clinical characteristics such as right heart catheterization hemodynamics, 6MWT distance, and oxygen requirements after parenteral treprostinil initiation. A marginal homogeneity test was used to assess the statistical significance of echocardiogram parameters based on the severity of right ventricular dilation and dysfunction before and after treprostinil initiation. These data ranged from 36 days to 962 days.

## 3. Results

1. Demographics;
2. ILD-subtype and pulmonary function test;
3. Treatment Types;
4. Tolerability, Dosages and Adverse Effects;
5. Right Heart Catheterization Results;
6. Echocardiograph Results;
7. VA-ECMO and Lung Transplantation.

### 3.1. Demographics

Of the 14 patients, eight were male and six were female. Ethnic groups included Caucasian (42.85%), African American (35.7%), Asian/Indian (14.28%) and Hispanic (7.14%). The average age of the patients was 62 years at the time of initial right heart catheterization. The mean BMI was 29.35 kg/m^3^. All patients had ILD diagnosed by Chest Computerized Tomography (CT) scan and pulmonary function tests (PFTs).

### 3.2. ILD–Subtype and Pulmonary Function Test

Within this group, patients were categorized by ILD-subtype. Six patients (42.85%) had connective tissue disorder associated with interstitial lung disease, two patients (14.28%) had ILD caused by sarcoidosis, three (21.43%) had idiopathic pulmonary fibrosis, and two (14.28%) had combined pulmonary fibrosis and emphysema (CPFE). One patient (7.14%) had Langerhans cell histiocytosis (Table 1).

The average percent predicted values for FVC and FEV1 were 63.21% and 60.93% respectively, with an average FEV1/FVC ratio of 77.99%. The average total lung capacity (TLC) was 3.77 +/− 1.11 L, the average residual volume (RV) was 1.57 +/− 0.47 L and the average DL_CO_ was 31.09 +/− 11.86%. This describes a subset population with a moderate restrictive pattern, without underlying airway obstruction, and with severe diffusion impairment on pulmonary function test. The average 6MWT distance was 201.8 m at initial right heart catheterization compared with 203.8 m after parenteral treprostinil initiation. Exertional oxygen requirements measured during the 6MWT did not worsen after the initiation of parenteral treprostinil (Table 2).

### 3.3. Treatment Types

In addition to parenteral treprostinil, all patients were on additional pulmonary hypertension therapies. Thirteen patients (92.8%) were on phosphodiesterase-5 (PDE-5) inhibitors, either sildenafil or tadalafil. No patients were on endothelin receptor antagonist; two patients (14.28%) were previously on inhaled prostacyclins, which were discontinued when parenteral treprostinil was initiated. Four patients were on antifibrotic therapy. Five patients were on anti-inflammatory therapies other than steroids that included mycophenolate (35.6%), rituximab (7.1%), and azathioprine (7.1%).

### 3.4. Tolerability, Dosages and Adverse Effects

Overall, parenteral treprostinil was well tolerated. Patient-reported adverse effects included headache, flushing, diarrhea and irritation at the site of administration specifically for subcutaneous treprostinil. If a patient experienced adverse effects while on parenteral treprostinil, the rate of titration was slowed. One patient experienced a line-associated bacteremia when first initiated on intravenous treprostinil. The patient was treated with intravenous antibiotics and transitioned to subcutaneous treprostinil upon discharge.

### 3.5. Right Heart Catheterization Results

Baseline hemodynamic parameters obtained via right heart catheterization demonstrated severe pre-capillary pulmonary hypertension (Table 2). Out of the fourteen patients, nine had repeat heart catheterizations for comparison after the initiation of parenteral treprostinil. The remaining five patients did not undergo a follow-up catheterization because they received a lung transplant prior to repeat right heart catheterization; the time from initial RHC to lung transplantation for this group ranged from 19 to 167 days, with the average time being 80 days from the initial RHC to lung transplantation. Consequently, they were excluded from the paired statistical analysis. The time to repeat right heart catheterization for the nine patients included in the paired analysis ranged from 38 days to 31 months, with the average time to repeat catheterization being 17 months.

**Figure 1:**
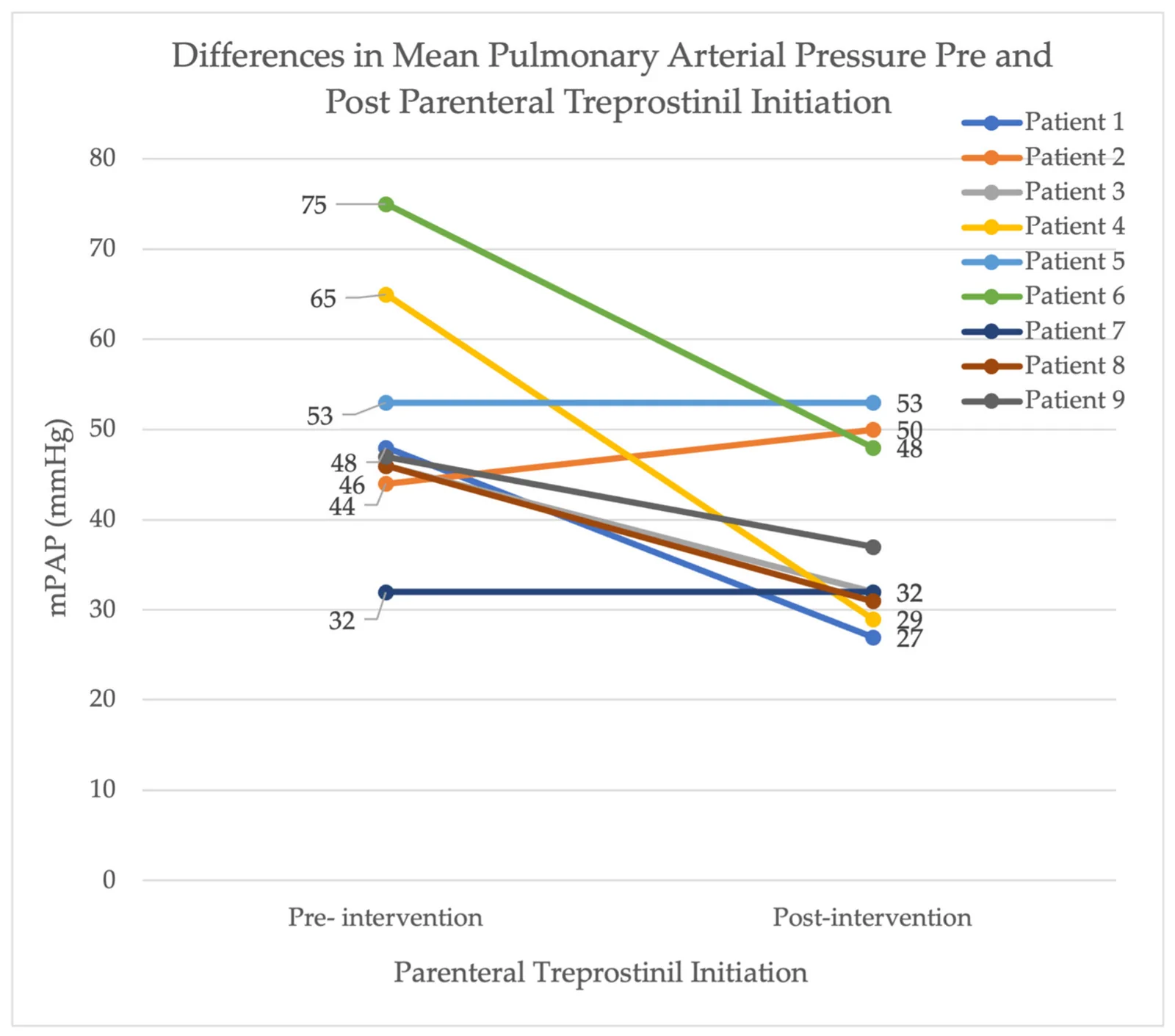
Differences in Mean Pulmonary Arterial Pressure (mPAP) Pre- and Post-Parenteral Treprostinil Initiation for nine patients with repeat right heart catheterizations.

Changes in matched hemodynamic parameters evaluated using the Wilcoxon signed-rank test are detailed in Table 2. The median baseline right atrial (RA) pressure decreased from 7.0 mmHg (IQR: 6.0–7.0) to 4.0 mmHg (IQR: 3.0–7.0) following parenteral treprostinil initiation (*p* = 0.176). Pulmonary artery systolic pressure (PASP) demonstrated a strong downward trend from a median baseline of 77 mmHg (IQR: 72–81) to 62 mmHg (IQR: 46–83), though it did not reach statistical significance (*p* = 0.055). However, mean pulmonary artery pressure (mPAP) demonstrated a statistically significant reduction, decreasing from a median baseline of 47.0 mmHg (IQR: 46.0–53.0) to 32.0 mmHg (IQR 31.0–48.0; *p* = 0.028; Figure 1). The median pulmonary capillary wedge pressure (PCWP) remained stable, shifting from 10.0 mmHg to 7.0 mmHg following treprostinil initiation (*p* = 0.547).

**Figure 2:**
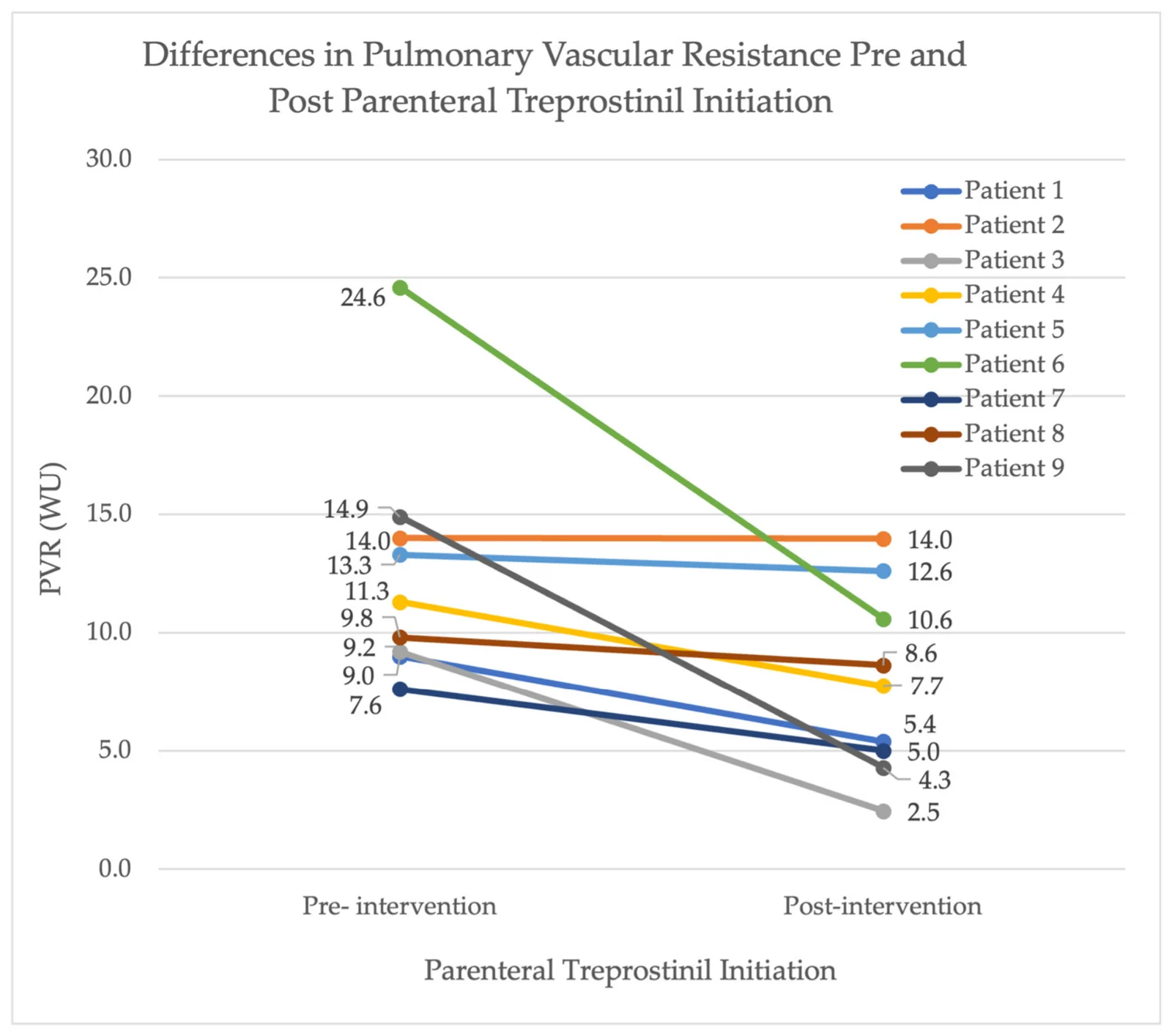
Differences in pulmonary vascular resistance (PVR) Pre- and Post-Parenteral Treprostinil Initiation for nine patients with repeat right heart catheterizations.

Cardiovascular performance demonstrated stable upward trends after the initiation of parenteral treprostinil. Median baseline cardiac output (CO) increased from 4.09 L/min to 4.65 L/min while median cardiac index increased from 1.95 L/min/m^2^ compared to 2.53 L/min/m^2;^ however, these values did not reach statistical significance (*p* = 0.25 for both). Pulmonary vascular resistance (PVR) improved significantly after parenteral treprostinil initiation from 11.3 Woods units (WU) (IQR: 9.2–14.0) to 7.8 WU (IQR: 5.0–10.6) (*p* = 0.012) (Figure 2). Additionally, median B-type natriuretic peptide (BNP) decreased from 320.0 pg/mL prior to parenteral treprostinil to 62.7 pg/mL following treatment (*p* = 0.009).

### 3.6. Echocardiography Results

Twelve patients (85.7%) underwent repeat transthoracic echocardiography(TTE) following the initiation of intravenous treprostinil. Of these patients, three patients had no evidence of right ventricular dilation or dysfunction. Seven patients had both right ventricular dilation and dysfunction, while one patient had RV dilation without RV dysfunction and one patient had isolated RV dysfunction without dilation.

Among patients with RV dilation, severity was classified as mild in two patients, moderate in four patients, and severe in two patients. Similarly, RV dysfunction was graded as mild in two patients, moderate in four patients, and severe in two patients.

Follow-up echocardiography demonstrated a reduction in RV dilation after the initiation of parenteral treprostinil. Five patients showed improvement in RV dilation severity; however, this trend did not reach statistical significance (*p* = 0.052). Comparison of pre- and post-treatment RV dysfunction using the marginal homogeneity test demonstrated no significant change following intervention (*p* = 0.21).

### 3.7. VA-ECMO and Lung Transplantation

Eight (57%) patients received a lung transplant, three of whom required VA-ECMO support as a bridge to transplantation. One of the three only required VA-ECMO support during transplant surgery due to their severe pulmonary hypertension and was decannulated immediately post-operatively. The median time to lung transplantation from initial right heart catheterization was 201 days (approximately 6.6 months). Two patients died prior to lung transplantation, one of whom remained active on the transplant waitlist for over two years and the other for just shy of one year. Of the two patients, the time from initiation of parenteral treprostinil to death was two years and eleven months, respectively. The cause of death was unknown for one patient. The second patient died from worsening respiratory failure and cardiogenic shock due to acute-on-chronic RV failure requiring VA-ECMO but was unable to undergo transplantation prior to death. Four patients are still active on the lung transplant list.

## 4. Discussion

Among patients with severe pulmonary hypertension associated with interstitial lung disease, the initiation of parenteral treprostinil as a bridge therapy to lung transplantation was associated with significant improvements in several key hemodynamic parameters. Notably reductions were observed in pulmonary artery systolic pressure, mean pulmonary artery pressure (mPAP), pulmonary vascular resistance (PVR) and BNP levels without evidence of worsening oxygenation. These data suggest that parenteral treprostinil therapy may serve as a valuable adjunctive therapy in carefully selected patients with advanced PH-ILD, particularly those awaiting lung transplantation.

### 4.1. Safety Profile and Ventilation-Perfusion Mismatch

Historically, the implementation of systemic, non-inhaled prostacyclin analogs in Group 3 pulmonary hypertension has been limited by concerns regarding systemic vasodilation, ventilation-perfusion (V/Q) mismatch, and subsequent hypoxemia [3,5,7]. A retrospective cohort study by Hinkamp et al. (2021), evaluated the use of parenteral prostacyclin in patients with severe Group 3 pulmonary hypertension and RV dysfunction [8]. Among the nine patients observed, there was no statistically significant improvement in NT-proBNP, 6 min walk distance, or WHO functional class after initiation of the medication and further worsened oxygenation in this group, a decline attributed to exacerbated V/Q mismatch [8].

In contrast, our findings suggest that parenteral treprostinil therapy can be both effective and safe from an oxygenation standpoint in select PH-ILD patients with severe pulmonary hemodynamics mimicking group 1 PAH physiology. This aligns with a similarly sized study published in 2014, which demonstrated that parenteral treprostinil improved right heart hemodynamics and echocardiographic function without affecting oxygen saturation, specifically in patients with advanced pulmonary hypertension and pulmonary fibrosis [9]. Notably, the severity of right heart hemodynamics was slightly less pronounced, with an mPAP of 47 mmHg compared with greater than 50 mmHg in our population. Consequently, this study provides important evidence that parenteral treprostinil can be safely used for pulmonary fibrosis associated with pulmonary hypertension as a bridge to transplantation without exacerbating ventilator-perfusion mismatch. For our study cohort, patients were started on parenteral treprostinil therapy as opposed to inhaled treprostinil therapy at the discretion of the treating pulmonologist due to their severe group 1 PAH physiology and the majority being too decompensated to expect significant benefit from inhaled therapy alone.

### 4.2. Pulmonary Hemodynamics and RV Afterload Reduction

In our study, pulmonary capillary wedge pressure remained stable throughout treatment, suggesting that the observed hemodynamic improvements were primarily related to reductions in pulmonary vascular load or blood flow across the lung itself rather than changes in left-sided filling pressures. The potential benefits of parenteral treprostinil in end-stage PH-ILD are likely multifactorial. By reducing pulmonary vascular resistance and right ventricular afterload, treprostinil may improve RV performance, enhance functional status, and promote hemodynamic stabilization in patients awaiting transplantation [9].

### 4.3. Vascular Phenotypes and Pulmonary Functions Analysis

Baseline pulmonary function tests demonstrated a moderate restrictive ventilatory defect without underlying airway obstruction alongside a severe reduction in diffusing capacity. The significant reduction in gas exchange efficiency, with DLco averaging 31%, is disproportionately severe compared to the moderate loss of lung volumes, representing advanced alveolar-capillary membrane destruction and early pulmonary vascular remodeling.

Our case review demonstrated therapeutic benefit and safety across a heterogeneous group of PH-ILD patients. Only three patients had IPF. Almost half of these patients had underlying connective tissue disease (CTD), which is a well-established risk factor for group 1 PAH physiology [9]. In patients with CTD-ILD, pulmonary vascular remodeling and PAH may progress much more aggressively compared to classic IPF groups. This pathophysiologic split mirrors findings in the sarcoidosis-associated pulmonary hypertension literature, where early, aggressive treatment using PAH-targeted therapies delays the progression of right ventricular failure. Specifically, the early initiation of parenteral prostacyclin in systemic sclerosis-associated PH-ILD may optimize outcomes by targeting vascular remodeling before fibrosis and capillary bed destruction occur [10].

Importantly, however, we observed severe PAH physiology and a response to PH treatment even among those with other non-CTD-ILD subtypes who lacked obvious risk factors for WHO Group 1 PAH physiology. This further highlights a potential expanded role of parenteral prostacyclin in select cases of PH-ILD with severe hemodynamics with decompensated right heart failure and NYHA Class IV symptoms.

### 4.4. The Role of Mechanical Circulatory Support as a Bridge to Transplantation

Parenteral treprostinil may offer substantial clinical benefit as an add-on therapy for transplant waitlist patients who experience severe hemodynamic instability, the need for critical care, or VA-ECMO support. Furthermore, in patients presenting with acute RV failure, parenteral treprostinil has been shown to be a successful stabilization method prior to transitioning to inhaled prostacyclin therapies for long-term maintenance [11]. Notably, within this high-risk cohort, only two patients died prior to lung transplantation, one of whom was safely maintained and survived for two years while receiving parenteral treprostinil therapy.

Although the use of VA-ECMO was not eliminated by parenteral treprostinil, survival to transplantation in most of these patients suggests a potential role as a bridge to transplantation in addition to parenteral prostacyclin therapy in this high-risk population. In these patients, severe right ventricular dysfunction is common, and VA-ECMO should not be delayed when clinically indicated despite prostacyclin therapy. These findings highlight the complexity of managing patients with end-stage PH-ILD and the evolving institutional practices and availability of ECMO support while emphasizing the importance of multidisciplinary care and coordination between pulmonary hypertension, transplant, critical care, and ECMO teams.

While patients with a specific severe phenotype of PH-ILD are awaiting transplantation, the addition of parenteral prostacyclin therapy serves as an effective strategy for pre-transplant stabilization and symptom mitigation. Although a definitive, generalized conclusion cannot be drawn from this small retrospective case series, our data emphasize that meticulous clinical monitoring is essential during the initiation and titration of this drug. Further large-scale, prospective studies are still required to determine whether parenteral treprostinil can improve long-term outcomes, including mortality, waitlist survival, PVR, right ventricular function, oxygen requirements, pulmonary function tests and six-minute walk test results.

### 4.5. Study Limitations

The limitations of this study include its retrospective case series design, small sample size, and the absence of a standardized protocol for parenteral treprostinil initiation, titration, and follow-up. The retrospective nature of this chart review restricted our ability to ensure consistent monitoring and made systematic routine follow-up difficult. Additionally, patients were selected for parenteral treprostinil based on clinical judgment, introducing the potential for selection bias and limiting the generalizability of these findings.

## 5. Conclusions

In conclusion, this retrospective case series demonstrates that parenteral treprostinil can serve as a safe and effective bridge therapy to lung transplantation in a carefully selected subpopulation of patients with advanced PH-ILD. Notably, the initiation of therapy led to significant improvements in key right heart hemodynamic parameters and biomarkers without provoking ventilation-perfusion mismatch or clinical hypoxemia. Furthermore, the high rates of survival to transplantation observed in this cohort suggest a valuable role for advanced pulmonary arterial hypertension therapies alongside mechanical circulatory support when appropriate.

While patients with a specific, severe phenotype of PH-ILD are awaiting transplantation, the addition of parenteral treprostinil therapy serves as an effective strategy for pre-transplant stabilization and symptom mitigation. Although a definitive, generalized conclusion cannot be drawn from this small retrospective case series, our findings add to the limited literature regarding the use of parenteral treprostinil and support its use as an adjunctive therapy in selected patients with advanced PH-ILD and severe hemodynamic impairment.

Given the complexities of managing end-stage PH-ILD, these findings emphasize the critical need for multidisciplinary coordination among pulmonary hypertension, transplant, and critical care teams. While this single-center experience provides encouraging real-world evidence for pre-transplant stabilization, larger multi-center prospective studies and randomized controlled trials are warranted to define optimal patient selection criteria, establish standardized titration protocols, and evaluate long-term outcomes in this high-risk population.

## Figures and Tables

**Table 1:** A total of fourteen patients were included in this study; the characteristics of the group are summarized below. Numbers reported are mean +/− SD.

Demographics (*N* = 14)	
Age, years	62 +/− 7.17
Range, years	43–70
Sex, *n* (%)	
Male	8 (57.1%)
Female	6 (42.85%)
BMI, kg/m^3^	29.35 +/− 5.69
Ethnicity, *n* (%)	
Caucasian	6 (42.85%)
African American	5 (35.7%)
Asian/Indian	2 (14.28%)
Hispanic	1 (7.14%)
Other	0 (0%)
ILD subtype, %	
Connective Tissue Disease ILD	42.85%
Idiopathic Pulmonary Fibrosis	21.43%
Sarcoidosis-associated ILD	14.28%
Combined Pulmonary Fibrosis and Emphysema	14.28%

**Table 2:** Comparison between pre- and post-treatment right heart catheterization hemodynamics, echocardiographic parameters, and six-minute walk test results following parenteral treprostinil initiation. Data are presented as medians (25th–75th percentiles). *p*-values were calculated using Wilcoxon’s signed-rank test.

Hemodynamic Parameter	Pre-Treatment (*n* = 14)	Post-Treatment (*n* = 9)	*p*-Value
RA Pressure, mmHg	7.0 (6.0–8.8)	4.0 (3.0–7.0)	0.176
Systolic PAP, mmHg	77.0 (72.0–81.0)	62.0 (46.0–83.0)	0.055
Mean PAP (mPAP), mmHg	47.0 (46.0–53.0)	32.0 (31.0–48.0)	0.028 *
PCWP, mmHg	10.0 (8.0–12.0)	7.0 (6.0–10.0)	0.574
Cardiac Output, L/min	4.09 (3.23–4.59)	4.65 (3.90–5.00)	0.250
Cardiac Index, L/min/m^2^	1.95 (1.77–2.44)	2.53 (2.03–2.70)	0.250
PVR, WU	11.3 (9.2–14.0)	7.7 (5.0–10.6)	0.012 *
**Echocardiograph Parameters (*N* = 14, 13)**			
RV dilation			0.052
No Evidence of RV dilation	0 (0%)	4 (30.7%)	
Mild dilation	2 (14.3%)	2 (15.4%)	
Moderate Dilation	4 (28.5%)	3 (23.1%)	
Severe Dilation	8 (57.1%)	4 (30.7%)	
RV Dysfunction			0.210
No Evidence of RV Dysfunction	4 (28.5%)	5 (38.4%)	
Mild Dysfunction	1 (7.1%)	2 (15.4%)	
Moderate Dysfunction	5 (35.7%)	5 (38.4%)	
Severe Dysfunction	4 (28.5%)	1 (7.8%)	
6 min walk test results			
6 min walk test distance, m	195.0 (150.0–255.0)	215.5 (188.2–239.0)	0.770
Oxygen Requirement, lpm	8.6	9.1	0.714
B-type natriuretic peptide (BNP), pg/mL	320 (106.3 to 638.5)	62.7 (25.9 to 210.1)	0.0085 **

* *p*-value < 0.05, ** *p*-value < 0.005. RA = Right atrial, PAP = pulmonary arterial pressure, PCWP = pulmonary capillary wedge pressure, PVR = pulmonary vascular resistance, WU = Woods Units, RV = right ventricular.

## Data Availability

The raw data supporting the conclusions of this article will be made available by the authors on request.

## Abbreviations

The following abbreviations are used in this manuscript:

PH-ILD	Pulmonary Hypertension-associated Interstitial Lung Disease
VA-ECMO	Veno-arterial extracorporeal membrane oxygenation
PAH	Pulmonary Arterial Hypertension
NYHA	New York Heart Association
IV	Intravenous
RHC	Right Heart Catheterization
RA	Right atrial
PAP	Pulmonary Arterial Pressure
PCWP	Pulmonary Capillary Wedge Pressure
PVR	Pulmonary Vascular Resistance
WU	Woods Units
RV	Right Ventricular or Right Ventricle

## References

[1] Barnes H., Yeoh H.L., Fothergill T., Burns A., Humbert M., Williams T. (2019). Prostacyclin for pulmonary arterial hypertension. Cochrane Database Syst. Rev..

[2] Barst R.J., Rubin L.J., Long W.A., McGoon M.D., Rich S., Badesch D.B., Groves B.M., Tapson V.F., Bourge R.C., Brundage B.H. (1996). A comparison of continuous intravenous epoprostenol (prostacyclin) with conventional therapy for primary pulmonary hypertension. N. Engl. J. Med..

[3] Waxman A., Restrepo-Jaramillo R., Thenappan T., Ravichandran A., Engel P., Bajwa A., Allen R., Feldman J., Argula R., Smith P. (2021). Inhaled Treprostinil in pulmonary hypertension due to interstitial lung disease. N. Engl. J. Med..

[4] Taichman D.B., Ornelas J., Chung L., Klinger J.R., Lewis S., Mandel J., Palevsky H.I., Rich S., Sood N., Rosenzweig E.B. (2014). Pharmacologic therapy for pulmonary arterial hypertension in adults: CHEST guideline and expert panel report. Chest.

[5] Olschewski H., Ghofrani H.A., Walmrath D., Schermuly R., Temmesfeld-Wollbruck B., Grimminger F., Seeger W. (1999). Inhaled prostacyclin and iloprost in severe pulmonary hypertension secondary to lung fibrosis. Am. J. Respir. Crit. Care Med..

[6] Olive J.K., Yaport M., Al-Qudsi O., Cutrone M., Abramson L., Ledbetter L., Alvin A., Vatsaas C.J., Milano C.A., Bronshteyn Y.S. (2025). A Scoping Review of Validated Echocardiographic Methods for Grading Right Ventricular Dysfunction: Proposal for an Evidence-Based Multiparametric Framework. J. Cardiothorac. Vasc. Anesth..

[7] Skoro-Sajer N. (2012). Optimal use of treprostinil in pulmonary arterial hypertension: A guide to the correct use of different formulations. Drugs.

[8] Hinkamp C.A., Shah T., Bartolome S., Torres F., Chin K.M. (2021). Parenteral prostanoids for severe Group 3 pulmonary hypertension with right ventricular dysfunction. J. Thorac. Dis..

[9] Saggar R., Khanna D., Vaidya A., Derhovanessian A., Maranian P., Duffy E., Belperio J.A., Weigt S.S., Dua S., Shapiro S.S. (2014). Changes in right heart haemodynamics and echocardiographic function in an advanced phenotype of pulmonary hypertension and right heart dysfunction associated with pulmonary fibrosis. Thorax.

[10] Volkmann E.R., Saggar R., Khanna D., Torres B., Flora A., Yoder L., Clements P.J., Elashoff R.M., Ross D.J., Agrawal H. (2014). Improved transplant-free survival in patients with systemic sclerosis-associated pulmonary hypertension and interstitial lung disease. Arthritis Rheumatol..

[11] Provencher S., Mai V., Bonnet S. (2024). Managing Pulmonary Arterial Hypertension with Cardiopulmonary Comorbidities. Chest.

